# Rapid and Reversible Development of Axonal Varicosities: A New Form of Neural Plasticity

**DOI:** 10.3389/fnmol.2021.610857

**Published:** 2021-02-03

**Authors:** Chen Gu

**Affiliations:** Department of Biological Chemistry and Pharmacology, The Ohio State University, Columbus, OH, United States

**Keywords:** axonal varicosity, mild traumatic brain injury, Alzheimer's disease, action potential, synaptic transmission, neural plasticity, mechanosensitive ion channel, microtubule

## Abstract

Axonal varicosities are enlarged, heterogeneous structures along axonal shafts, profoundly affecting axonal conduction and synaptic transmission. They represent a key pathological feature believed to develop via slow accumulation of axonal damage that occurs during irreversible degeneration, for example in mild traumatic brain injury (mTBI), Alzheimer's and Parkinson's diseases, and multiple sclerosis. Here this review first discusses recent *in vitro* results showing that axonal varicosities can be rapidly and reversibly induced by mechanical stress in cultured primary neurons from the central nervous system (CNS). This notion is further supported by *in vivo* studies revealing the induction of axonal varicosities across various brain regions in different mTBI mouse models, as a prominent feature of axonal pathology. Limited progress in understanding intrinsic and extrinsic regulatory mechanisms of axonal varicosity induction and development is further highlighted. Rapid and reversible formation of axonal varicosities likely plays a key role in CNS neuron mechanosensation and is a new form of neural plasticity. Future investigation in this emerging research field may reveal how to reverse axonal injury, contributing to the development of new strategies for treating brain injuries and related neurodegenerative diseases.

## Introduction

Neural plasticity is the ability of the central nervous system (CNS) to undergo biological changes, functionally and structurally, in response to stimuli (experience, injury, etc.). These changes range from the cellular level to the network and system levels. Neurons are the key structural and functional units in the CNS. They are highly polarized cells, and typically contain multiple dendrites and a single long axon, crucial for conveying input and output electrical signals, respectively. Communications between two neurons are normally carried out through one or more synapses where the plasma membrane of axonal terminal of the pre-synaptic neuron comes into close apposition with the dendritic membrane of the postsynaptic neuron. Specific patterns of synaptic activity can induce changes in synaptic strength, which is defined as synaptic plasticity and thought to contribute to learning and memory. Synaptic plasticity regulated by pre-synaptic or postsynaptic mechanisms is the major form of neural plasticity and has been extensively investigated. In contrast, other forms of adaptive changes in neurons have not attracted much attention, due to perhaps being less impactful on CNS functions compared to synaptic plasticity or simply being ignored. In particular, little is known about mechanical regulation of morphological and functional polarity of CNS neurons.

Besides chemical and electrical communications, neurons physically interact with their microenvironment constantly. The interaction depends on the forces acting on and exerted by the neuron, their mechanical properties, and coupling. Mechanosensation and mechanotransduction have been extensively investigated in specialized cell types or organs, including cochlear hair cells, kidney for fluid pressure (Arnadottir and Chalfie, 2010; Delmas et al., 2011; Nilius and Honore, 2012), but not in the brain, because the vertebrate brain is well-protected by the skull. Nonetheless, mechanical forces have been implicated in normal neural development, such as neurogenesis, neuron–glia interactions, neuronal migration, axonal outgrowth, growth cone motility, synapse and neural circuit formation, and brain folding (Van Essen, 1997; Gilmour et al., 2004; Engler et al., 2006; Lu et al., 2006; Elkin et al., 2007; Betz et al., 2011; Amack and Manning, 2012; Franze, 2013; Campas et al., 2014), but the underlying mechanisms are still poorly understood. This is because such investigation is often hindered by technical challenges in microbiomechanical measurements and manipulations, as well as a knowledge gap in signaling pathways of CNS mechanosensation.

One possible mechanism underlying CNS mechanosensation involves axonal varicosities (swellings or beadings). They are enlarged, heterogeneous structures along axonal shafts, profoundly affecting axonal conduction and synaptic transmission (Debanne, 2004). They represent a key pathological feature believed to develop via slow accumulation of axonal damage that occurs during irreversible degeneration, for example in mild traumatic brain injury (mTBI) and neurodegenerative diseases, including Alzheimer's disease (AD), Parkinson's disease (PD) and multiple sclerosis (Luo and O'Leary, 2005; Reeves et al., 2005, 2012; Browne et al., 2011; Toledo et al., 2012; Johnson et al., 2013; Yang et al., 2013). In the past, their initiation was thought very slow and irreversible. Our results published in 2017 showed that axonal varicosity formation induced by mechanical stress was unexpectedly rapid and partially reversible (Gu et al., 2017). In the same study, we further visualized their rapid induction in an mTBI mouse model, where the usage of Thy1-YFP transgenic mice was the key to clearly visualize such axonal change (Gu et al., 2017). Importantly, consistent with our results, there are several recent studies showing rather rapid induction of axonal varicosities *in vivo* in different types of mTBI mouse models (Marion et al., 2018; Vascak et al., 2018; Ziogas and Koliatsos, 2018; Pernici et al., 2019; Weber et al., 2019). Therefore, rapid and reversible formation of axonal varicosities in CNS neuron mechanosensation is likely a new form of neural plasticity that is related to mechanical injury.

### Axonal Varicosities in mTBI and Various Neurological Diseases

**Figure 1:**
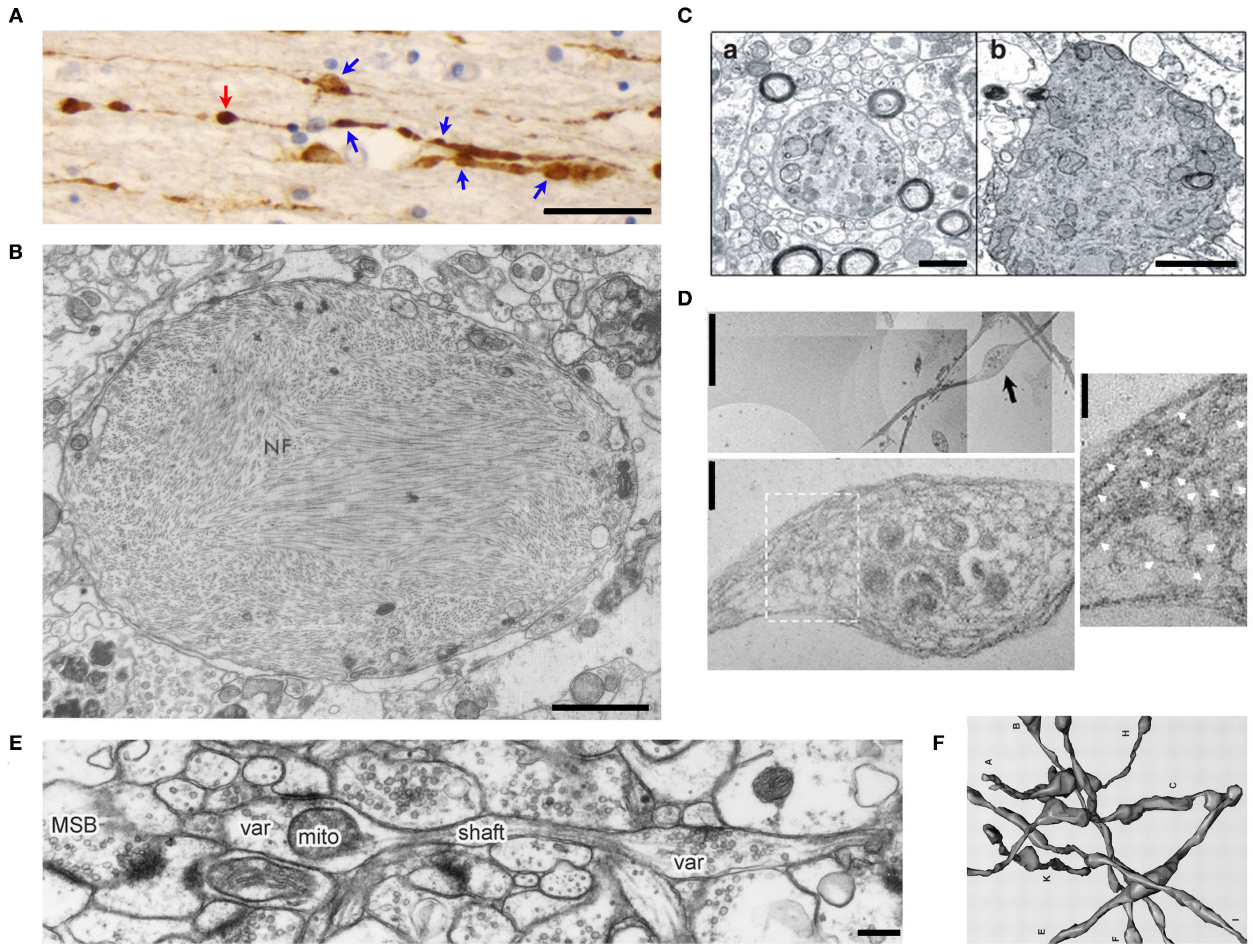
Morphology of axonal varicosities in injured, diseased or normal brains. **(A)**, Post-mortem APP immunoreactivity within the corpus callosum of a TBI patient displayed multiple varicosities (blue arrows) along individual axons (Tang-Schomer et al., 2012). The swelling without a visible thin linking axon is a potential terminal bulb (red arrow). Scale bar, 30 μm. **(B)**, The transmission electron microscope (TEM) image of an axonal varicosity or swelling filled with neurofibrils (NF) from the post-mortem cerebral cortex of a patient with Alzheimer's presenile dementia (Terry et al., 1964). Scale bar, 1 μm. **(C)**, Ultrastructure of axonal varicosity in a mouse model of AD (Stokin et al., 2005). Degenerative changes in axonal varicosity along the fibers of the nucleus basalis of Meynert in Tg-swAPPPrp (b) but not wild-type mice (a). Scale bar, 1 μm. **(D)**, TEM images of cultured cortical neurons from spastin^−/−^ mice show disorganization of the microtubule (MT) network within axonal varicosities (Fassier et al., 2013). The image in the lower panel is the high magnification of axonal varicosity indicated by a blackarrow in the upper image. The image on the right is the high magnification of boxed regions, showing the tangled and bent aspect of MT filaments within the axonal varicosity. This abnormal appearance of MTs was never observed in wild-type axons, in which MTs were always organized in parallel arrays. White arrows indicate MT. Scale bars: 5 μm (upper left), 0.5 μm (lower left) and 0.25 μm (right). **(E)**, Ultrastructural features of CA3 varicosities (Var) and axons in stratum radiatum of area CA1 in the normal rat brain (Shepherd and Harris, 1998). Longitudinally sectioned axon with two boutons, neither of which had additional PSDs or mitochondria in adjacent images. *mito*, mitochondria; *MSB*, multiple-synapse bouton; *PSD*, postsynaptic density. Scale bar, 1 μm. **(F)**, Eight reconstructed axons from series electron microscopy images including axonal varicosities (Shepherd and Harris, 1998).

Axonal varicosity is a key pathological hallmark in the brain suffered from mechanical injury. TBI is a leading cause of morbidity and mortality across the world. There were ~27 million new cases of TBI worldwide in 2016 (James et al., 2019). TBI severity is commonly classified as mild, moderate or severe. mTBI is also referred to as concussion, defined as the result of the forceful motion of the head or impact causing a brief change in mental status (confusion, disorientation or loss of memory) or loss of consciousness for <30 min. Diffuse axonal injury (DAI) is the most common and important feature, and observed in mild to severe TBI (Povlishock, 1992; Smith and Meaney, 2000; Browne et al., 2011). DAI features include axonal varicosities and terminal bulbs. Varicosities appear like beads on a string, whereas terminal bulbs are disconnected enlarged endings in axons, indicating broken axons. These damaged axons can be labeled with various markers. In post-mortem APP (amyloid precursor protein) immunostaining of the corpus callosum, no signal is normally observed in a healthy human subject, whereas staining signals from TBI patients displayed a classic pattern of axonal varicosities—multiple individual varicosities along the length of an individual axon, as well as potential terminal bulbs without narrow axonal shaft linking them (Figure 1A) (Tang-Schomer et al., 2012). In the past, the development of axonal varicosities was thought very slow and irreversible, preceding the formation of terminal bulbs in broken or degenerated axons.

Axonal varicosities are also present in neurodegenerative diseases and their mouse models. AD is an irreversible, progressive brain disorder showing slow impairment of memory and thinking skills. Ultrastructural studies using electron microscopy showed various axonal pathologies including axonal varicosities filled with neurofibrils in the brain of human patients with AD (Figure 1B) (Terry et al., 1964). In 4-month-old TgswAPPPrp mice, a mouse model for AD, axonal varicosities that were positive or negative for choline acetyltransferase (ChAT) in the nucleus basalis of Meynert were analyzed by comparing with wild-type mice (Stokin et al., 2005). These varicosities contained large numbers of organelles and vesicles, and were not myelinated, nor associated with postsynaptic densities. A subset of varicosities displayed characteristics of axonal degeneration, including electron-dense granular axoplasm and a large amount of axoplasmic debris of tubular appearance (Figure 1C) (Stokin et al., 2005).

PD is characterized by a progressive decline in voluntary motor function that primarily results from the death of dopaminergic neurons in the substantia nigra. Accumulation of intracellular proteinaceous aggregates containing misfolded α-synuclein proteins precedes neuronal death (Dauer and Przedborski, 2003). Axonal pathology including varicosities formed due to loss of expression of microtubule proteins in cultured dopaminergic neurons derived from human pluripotent stem cells that harbor the pathogenic A53T (G209A) mutation in the α-synuclein gene (Czaniecki et al., 2019). Interestingly, forced expression of the microtubule binding protein (MAP1B) could rescue axonal neuropathology (Czaniecki et al., 2019). Thus, axonal varicosities are considered as an early sign of irreversible neurodegeneration in both AD and PD.

Spastic paraplegia 4 (SPG4) encodes the microtubule-severing protein spastin. Its mutations are linked to the most frequent form of hereditary spastic paraplegia (HSP), a heterogeneous group of genetic diseases characterized by degeneration of the corticospinal tracts (Fink, 2006). As a key feature for progressive axonal degeneration, axonal varicosities developed with disorganized microtubule filaments and hence impaired axonal transport in cortical neurons of SPG4 knockout mice (Figure 1D) (Fassier et al., 2013). Taken together, since axonal varicosities were considered merely as a subcellular structure preceding axon degeneration, early studies did not make a major effort to distinguish axonal varicosities from terminal bulbs, nor to understand the transition from a varicosity to a terminal bulb.

Axonal varicosity formation was also observed as an early event in Wallerian degeneration (WD), leading to neurodegeneration (Beirowski et al., 2010; Yang et al., 2013). WD is the rapid degeneration of the distal stump of a transected axon and an active self-destruction program. Several hours after transection, axons in dorsal root ganglion, optic nerve, spinal cord dorsal column, and corpus callosum all started to form varicosities in their distal stumps before fragmentation, only with subtle differences in their timing and varicosity distribution patterns (Beirowski et al., 2010; Yang et al., 2013). Mitochondrial potential loss and swelling were frequently observed accompanying axonal varicosity formation in this process (Sievers et al., 2003; Park et al., 2013). Mitochondrial dysfunction can lead to calcium overload in the axon, decrease in ATP production and increase in reactive oxygen species (ROS). Preventing mitochondrial dysfunction by pharmacological inhibiting or shRNA knocking down cyclophilin D, a functional component of the mitochondrial permeability transition pore, could significantly delay severed axons from undergoing degeneration in *ex vivo* and *in vitro* models (Barrientos et al., 2011). Of note, in WD, varicosities started to develop only after the axons were cut, which were already in an irreversible situation.

Sometimes axonal varicosities can actually recover, which was reported in a mouse model of multiple sclerosis (Nikic et al., 2011). Multiple sclerosis is an inflammatory demyelination disease of the CNS with unknown origin, but it is the consensus that immune-mediated axon damage responsible for permanent neurological deficits. *In vivo* imaging of Experimental Autoimmune Encephalomyelitis (EAE) model revealed focal axonal degeneration, which began with axonal varicosities and progressed to axon fragmentation (Nikic et al., 2011). It appeared that focal intra-axonal mitochondrial pathology was the earliest ultrastructural sign of damage, and it preceded changes in axon morphology (Nikic et al., 2011). Interestingly, some axonal varicosities were observed to spontaneously recover, suggesting that inflammatory axon damage might be spontaneously reversible and thus a potential target for therapy (Nikic et al., 2011).

### Some Pre-synaptic Boutons as One Type of Axonal Varicosities

Axonal varicosities are present at a low level in the normal CNS and often form during axonal pruning and synapse formation (Westrum and Blackstad, 1962; Shepherd and Harris, 1998; Luo and O'Leary, 2005). In hippocampal slices, serial electron microscopy was used to examine a nearly homogeneous population of CA3-CA1 axons in the middle of stratum radiatum of area CA1 (Figures 1E,F) (Shepherd and Harris, 1998). The oval-shaped varicosities varied greatly in both length and volume and were linked by narrow axonal shafts. Most of these varicosities were pre-synaptic boutons containing synaptic vesicles and colocalizing with postsynaptic markers (Shepherd and Harris, 1998). It remains to be determined whether this type of axonal varicosities—the enlarged pre-synaptic boutons that also appear like beads on a string—can be initiated by mechanical stress and be reversible. In the literature, axonal varicosities are sometimes used to refer to enlarged pre-synaptic boutons that have the key function of neurotransmitter release and indicate locations of synapses under normal physiological conditions. Taken together, axonal varicosities are defined by morphology and they are present in normal, diseased and injured brains. Although axonal varicosities of pre-synaptic boutons differ from the ones developed in mTBI or neurodegenerative diseases in terms of function, they may share some components in the signaling pathways responsible for their initiation, development and long-term fate.

### Axonal Varicosities Profoundly Affect Action Potential Propagation

**Figure 2:**
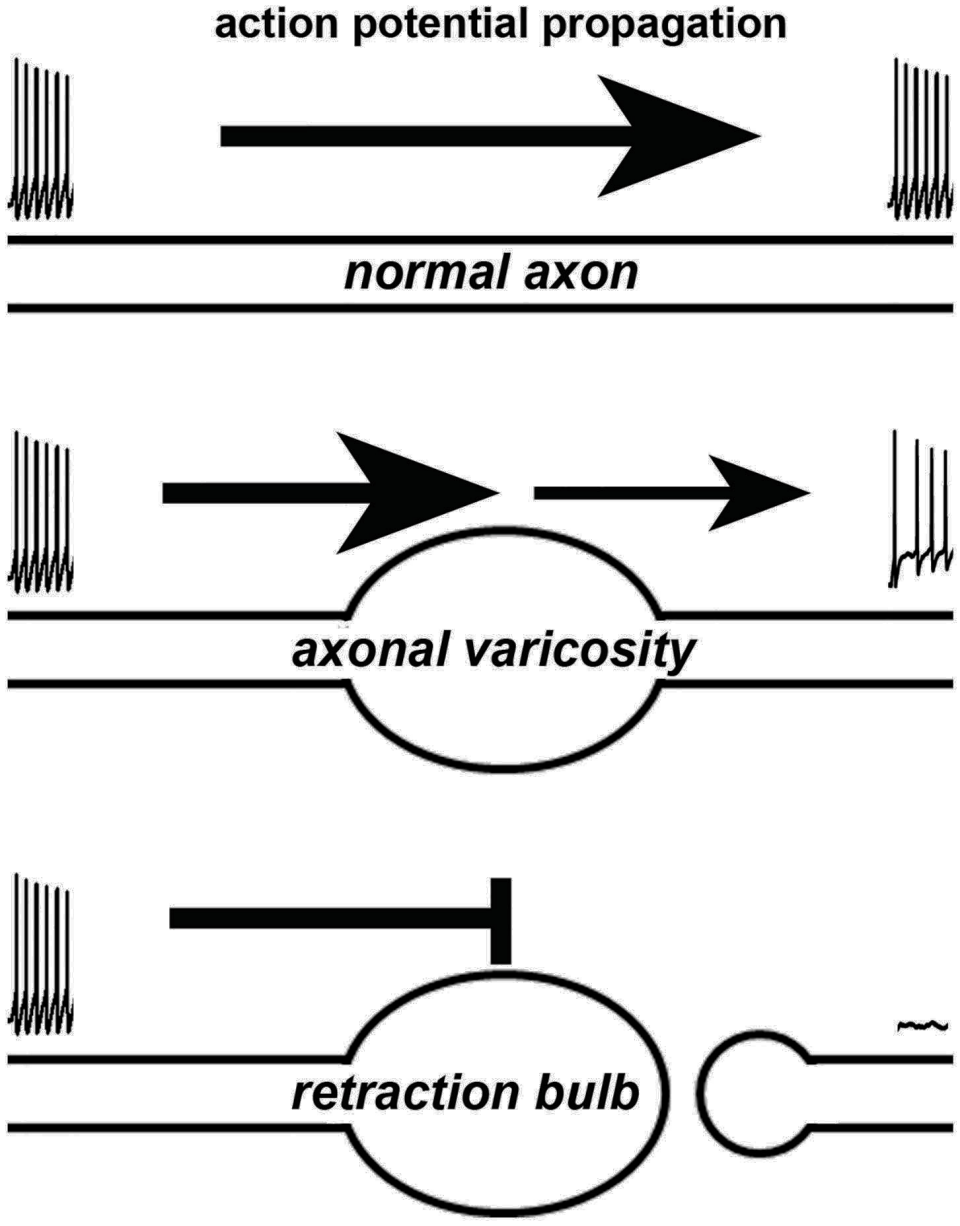
Effects of an axonal varicosity or a terminal bulb on action potential propagation along an axon. Action potentials propagate along a normal axon with high fidelity (top). Altered waveform, reduced frequency and even failure of action potentials can be induced by axonal varicosity (middle). A terminal bulb indicates a broken axon, which no longer allows action potentials to travel through.

How does a varicosity affect the axonal function? Axonal varicosities can induce failures of action potentials, when they propagate through the axon segment with abruptly increased diameter, revealed by electrophysiological recording, fast imaging and computer simulation (Figure 2) (Debanne, 2004). This is because increased axon diameter leads to a larger capacitance, which demands more incoming electrical currents to reach threshold membrane potential required for initiating an action potential through activating voltage-gated Na^+^ channels. It was reported that when action potentials propagated through a pre-synaptic bouton with increased axon diameter, failure could occur especially when there was an additional inhibitory conductance in rat posterior pituitary nerve terminals (Jackson and Zhang, 1995). Moreover, failure also could occur when action potentials propagated back to soma, where the diameter abruptly increased (Luscher et al., 1994; Antic et al., 2000; Evans et al., 2007). Thus, axonal varicosities that form under either normal or abnormal conditions can induce propagation failure of action potentials, similar to the failure of propagating through pre-synaptic boutons (a type of axonal varicosities) or back to soma. Furthermore, axonal varicosity, a kind of geometrical irregularities, can distort the waveform and velocity of propagating action potentials, as revealed by computer simulations (Luscher and Shiner, 1990; Manor et al., 1991). Failure can get worse in high frequency firing (Wu et al., 2020). On the other hand, axon terminal bulbs represent broken axons and directly eliminate axonal conduction of action potentials (Figure 2).

Besides the geometrical effect, local changes in ionic concentration or osmolarity, as well as availability of ion channels, can also affect action potential propagation through axonal varicosities. In rodent hippocampal mossy fiber boutons, there are very abundant voltage-gated Na^+^ channels to counteract the geometrical effects and increase the reliability and speed of action potential propagation (Engel and Jonas, 2005). Therefore, due to the heterogeneity of axonal varicosities in various diseases and injuries, their specific effects on action potential propagation still need to be experimentally determined under each condition.

### Axonal Varicosities Induced by Mechanical Stress in Cultured Neurons

**Figure 3:**
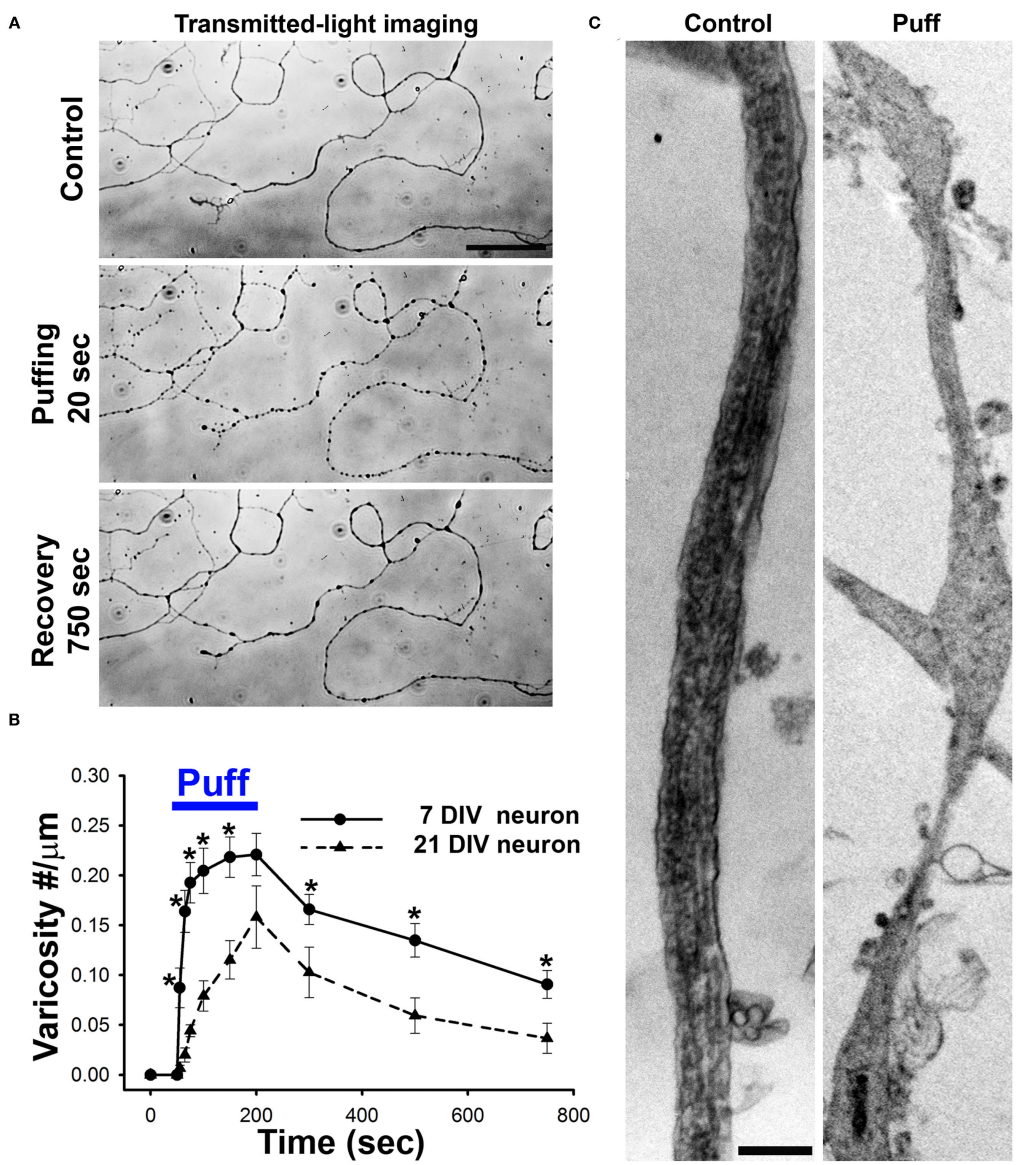
Axonal varicosities are rapidly and reversibly induced by mechanical stress in cultured CNS neurons. **(A)**, Puffing induced rapid and reversible formation of axonal varicosities in cultured hippocampal neurons at 7 days *in vitro* (DIV), revealed by microscopy with transmitted lights (Gu et al., 2017). Mechanical pressure was delivered by puffing of Hank's buffer via a glass pipette onto cultured neurons with 190 mm H_2_O static pressure at the tip for 150 s. Axonal varicosities slowly and partially recovered after puffing. **(B)**, Summary of puffing-induced varicosity densities along axons from younger (7 DIV) and older (21 DIV) neurons (Gu et al., 2017). **(C)**, TEM images of axonal segments under control condition (left) and when developing multiple varicosities induced by puffing (right) (Gu et al., 2017). Scale bars, 50 μm in **(A)** and 1 μm in **(C)**.

Since axonal varicosities are often associated with neurodegeneration, their initiation was thought very slow and irreversible in the past. Our results published in 2017 first showed that axonal varicosity formation induced by mechanical stress was unexpectedly rapid (within 5 s) and reversible (>20 min for half recovery) (Gu et al., 2017). We discovered this phenomenon by accident. In a control experiment for local drug perfusion, we found that puffing Hank's buffer itself without any drug induced rapid and reversible varicosity formation along axons of cultured hippocampal neurons (Figure 3). Importantly, the exact same solution from the same bench was used for both the puffing pipette and the bath each time. The speed and size of axonal varicosity formation correlated with the strength of puffing pressure (mainly out-of-plane compression). The minimal pressure that reliably induced axonal varicosities was determined to be ~0.25 ± 0.06 nN/μm^2^ onto cultured neurons at the center of puffing area, while the static pressure at the tip of puffing pipette was 190 mm H_2_O. The pressure value onto the neurons is well within the physiological/pathophysiological range in the brain (Gu et al., 2017). This experimental setting may mimic increased local mechanical stress onto unmyelinated CNS axons induced by cell growth and migration, tissue swelling or mechanical impact. Overall, this biomechanical assay system is highly reliable, produces highly repeatable results and is an ideal system for both (1) mechanistic studies of CNS neuron mechanosensation and injury, and (2) drug screen for discovering new therapeutic chemicals for treating mTBI or TBI (Servello et al., 2018).

Axonal varicosity formation induced by fluid mechanical stress has intriguing spatial-temporal features. Under the same level of stress, the varicosities were preferentially induced in middle and distal axons, but not in dendrites and proximal axons, and varicosity induction was also a highly localized event restricted within the axonal segment exposed to the puffing (Gu et al., 2017). In contrast, dendrites could develop varicosities under an excitotoxicity condition over a much longer period of time (~20 min) (Gu et al., 2017). This polarized formation of varicosities may result from specific subcellular distributions of a mechanosensitive ion channel TRPV4 (transient receptor potential cation channel subfamily V member 4) and microtubule-binding protein MAP6 (Gu et al., 2017). In our system, the sites of axonal varicosity induction were not the pre-synaptic boutons. Most of those induced axonal varicosities were initiated at smooth axonal shaft. Moreover, these puffing experiments mainly used cultured neurons from 7 to 11 days *in vitro* (DIV), while these neurons normally start to form synapses around 14 DIV (Gu et al., 2017).

Axonal varicosity induction by fluid puffing displayed two unique temporal features, rapidness and reversibility. The onset of axonal varicosity induction was <5 s for young neurons (7 DIV), and gradually increased when the neurons become more mature (Figure 3B). The 2-s-pulse puffing induced varicosity formation in a frequency-dependent manner (Gu et al., 2017). When puffing stopped, most varicosities could slowly recover (>20 min for half recovery) to different extents (Gu et al., 2017). Larger varicosities induced by longer or stronger puffing less likely recover completely. Based on our experimental results, including channel modulators, siRNA knockdown, imaging and immunostaining, we proposed a new model that micromechanical stress activates TRPV4 in axonal membranes, leading to Ca^2+^ influx into the axon. Subsequently, increased Ca^2+^-bound calmodulin levels in the axon compete with MAP6 for microtubules, which destabilizes microtubules and thus disrupts normal axonal transport, further leading to aberrant accumulation of axonal cargos and formation of axonal varicosities (Gu et al., 2017).

Axonal varicosity formation was also observed in other *in vitro* systems with cultured CNS neurons. Using a model of dynamic stretch injury, the Smith group examined axons spanning two populations of cultured cortical neurons (10–12 DIV) separated by lithographically-fabricated micro-channels received quantifiable uniaxial strain driven by pressured air (~100 ms total duration) (Smith et al., 1999; Yuen et al., 2009). Undulations were observed after stretch with at least 5% strain, whereas 75% strain caused immediate undulations, followed by the development of axonal varicosities by 3 h (Yuen et al., 2009; Tang-Schomer et al., 2012). Here undulations refer to wave-like axonal shafts that formed from a straight orientation right after being stretching. Physical breaks in the microtubule filaments followed by accumulation of transported materials in discrete swellings were proposed to contribute to axonal injury (Tang-Schomer et al., 2012).

The Parker group used two *in vitro* systems to mimic traumatic injury to rat cortical neurons, which included a high velocity stretcher and a magnetic tweezer (Hemphill et al., 2011). In the stretch model, after substrates underwent an abrupt, uniaxial stretch (at 1% per ms) to generate a strain field of defined magnitude, significant amount of axonal varicosity formation was observed (~10 min after stretch) only when the strain was larger than 10% (Hemphill et al., 2011). In the second system, magnetic tweezers were used to generate abrupt pulling forces (100 ms to 1 s; 0.5–5.5 nN) to poly-L-lysine- or fibronectin-coated paramagnetic beads attached to the surfaces of cultured neurons to effectively induce axonal varicosities (Hemphill et al., 2011). Here, axonal varicosities started to form from 5 to 10 min after pulling and some appeared to form retraction bulbs of broken axons (Hemphill et al., 2011). The authors further showed that Rho-kinase inhibitor but not calpain inhibitor significantly reduced axonal varicosity formation and axonal injury in response to either 5 or 10% strain, suggesting an important role of integrin-mediated activation of Rho in the process (Hemphill et al., 2011).

Using a model of transient axonal stretch injury, the Vickers group showed that a single blast of sterile air was used to deflect bundles of axons of cultured cortical neurons at 21 DIV, resulting in a transient 1–6% increase in original axon length (Chung et al., 2005). The axonal bundle quickly returned to original position afterwards. There was no obvious change in axonal bundles until 48 h post-injury, when these axons started to display increased neurofilament expression, bundle derangement, swelling and secondary axotomy (Chung et al., 2005). Pretreating the culture neurons with cyclosporin-A, an inhibitor of calcineurin and the mitochondrial membrane transitional pore, reduced cytoskeletal damage in this stretch-injured axonal bundles (Staal et al., 2007).

In the experimental setting of fluid puffing, the mechanical stress mainly consists of out-of-plane compression (Gu et al., 2017). Since there was no gross movement of neuronal processes in puffing experiments, there was no involvement of uniaxial stretching or bending (Gu et al., 2017). In stark contrast, other experimental settings that were discussed above mainly involved bending and uniaxial stretching (or tension), for instance, deflection of axonal bundles like moving strings (Chung et al., 2005; Staal et al., 2007) or stretching axons growing on micropatterned channels or stretchable membranes (Hemphill et al., 2011; Tang-Schomer et al., 2012). Although different types of mechanical stresses were involved, it is the consensus that all of these *in vitro* models can induce axonal varicosities. However, it is important to note that they displayed striking differences in the onset time and reversibility of axonal varicosity formation. Fluid puffing induced axonal varicosities within 5 s and partial reversibility (Gu et al., 2017), whereas it took much longer (from 10 min to several hours) to induce axonal varicosities in other systems, where the varicosities also appeared irreversible (Staal et al., 2007; Hemphill et al., 2011; Tang-Schomer et al., 2012). Therefore, different types of mechanical stresses appear to differ in their efficacy in axonal varicosity induction. Currently, the mechanism underlying the difference remains unknown. This is an interesting research topic for future investigation.

Mechanical stress-induced axonal varicosities can profoundly affect axonal functions. The geometrical changes alone may induce action potential failure and frequency change. Furthermore, other local changes in ionic concentration, osmolarity and ion channel targeting have not yet been understood, especially due to the heterogeneity of axonal varicosities. Besides its impact on orthodromic propagation of action potentials, the fluid-puffing study showed that axonal varicosity induction can trigger antidromic propagation of action potentials back to neuronal soma (Gu et al., 2017). This finding represents a novel and rapid axon–soma communication upon varicosity initiation mediated by back-propagating action potentials. Earlier studies showed that axonal injuries induce axon–soma communication mainly through the two mechanisms: a rapid one encoded by Ca^2+^ waves and a slower one conveyed by molecular motors (Ziv and Spira, 1995; Cho et al., 2013; Rishal and Fainzilber, 2014). Therefore, extensive investigations are still needed to understand the functional impact of induced axonal varicosities on neurons under different conditions.

### Axonal Varicosities Induced by Mechanical Impact *in vivo*

**Figure 4:**
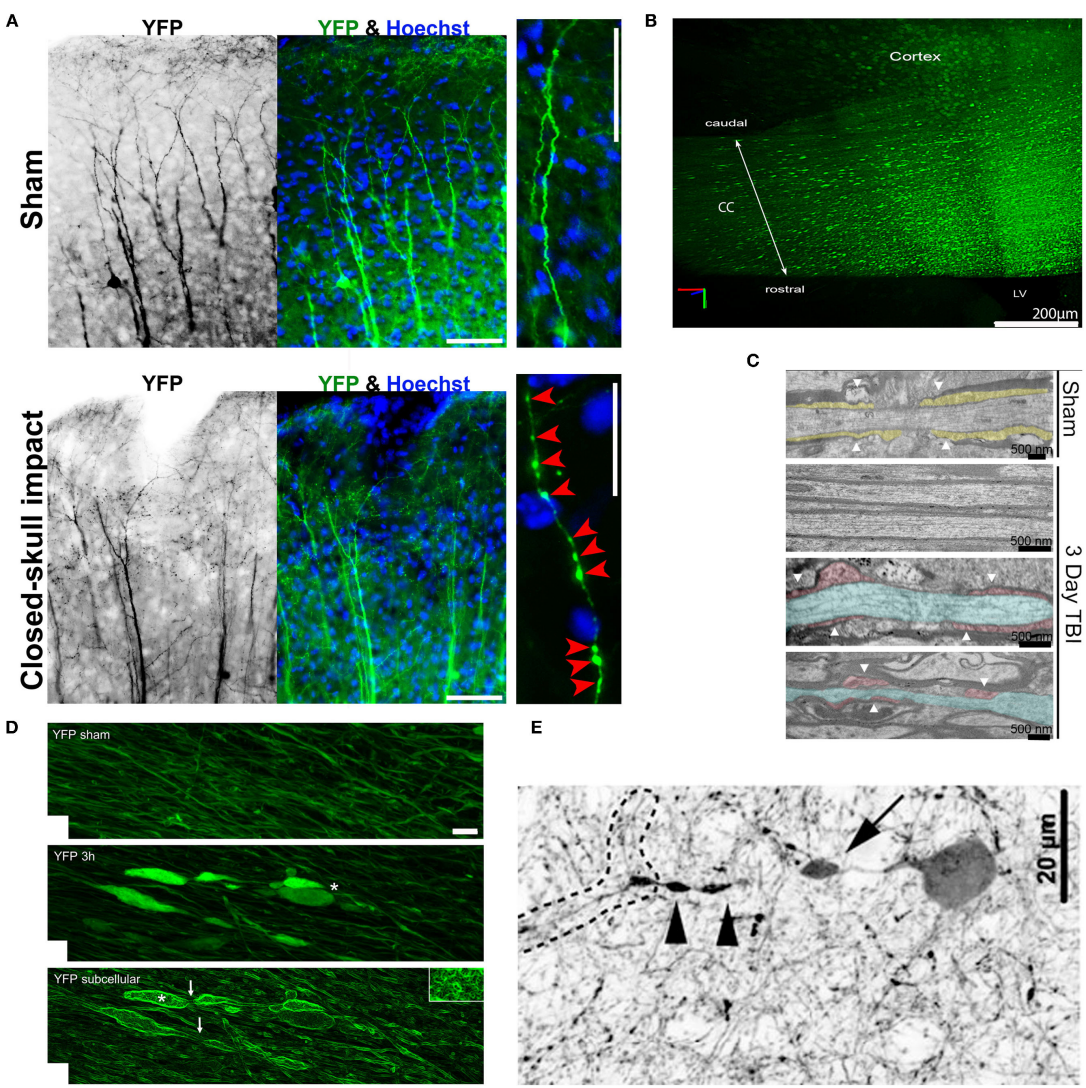
Axonal varicosities induced *in vivo* by mechanical impact in mouse models of mTBI. **(A)**, The repetitive closed-skull impact model induced axonal varicosities in multi-focal fashion in the cortex of Thy1-YFP transgenic mice (Gu et al., 2017). Mice were perfused and fixed immediately after the 2nd impact. Sham and impacted mice, upper and lower panels. YFP fluorescent signals are inverted in the gray scale image on the left. The apical dendrites of layer V projection neurons in the cortex point in the upward direction. Higher magnification images on the right show individual axons. Red arrowheads, induced axonal varicosities. Scale bars: 100 μm for left/middle panels and 20 μm for right panels. **(B)**, Concussive TBI (closed-skull) induced YFP+ axonal varicosities in the corpus callosum (CC) under the impact site illustrated using CLARITY (Marion et al., 2018). The mouse was sacrificed 3 days after impact. LV, lateral ventricle. **(C)**, TEM images of coronal section through the CC to illustrate organized myelin loop attachments forming paranodes (yellow fill; white arrowheads) in the sham (top), and axons in impacted mice with normal myelin and adjacent damaged axons (blue) with cytoskeletal breakdown paranodes (red fill) and myelin loss (bottom) (Marion et al., 2018). **(D)**, Axonal varicosities were induced in the corticospinal tract 3 h after weight-drop impact (Ziogas and Koliatsos, 2018). Confocal images of sham (top) and injured (middle with conventional confocal microscopy and bottom using edge-detection function. White asterisks, axonal terminal bulbs; White arrows, thin axons bridging swellings. Scale bar, 5 μm. **(E)**, A representative tdTomato+ interneuron showing a perisomatic axonal varicosity (arrow) and more distal varicosities (arrowheads) induced in a central fluid percussion model (Vascak et al., 2018).

Although axonal varicosities were observed in TBI brains, it was not known whether these varicosities formed in a direct response to mechanical impact or were indirectly induced by altered microenvironment. In repetitive closed-skull mTBI model (rcTBI) using Thy1-YFP transgenic mice, many axonal varicosities were found in the somatosensory cortex in a multifocal fashion immediately after the second impact (Figure 4A) (Gu et al., 2017). This was the first time that such early time point was examined in this rcTBI model (Gu et al., 2017). The YFP-containing axons and dendrites of the subset projection neurons can be clearly visualized in these transgenic mice, making the analysis possible without relying on the expression of pathological markers (i.e., APP or phospho-tau). These YFP+ axonal varicosities resemble those in cultured neurons induced by puffing in terms of the size, number, and pattern (Gu et al., 2017). To verify whether some of these varicosities in mTBI mice might be pre-synaptic boutons, two different endogenous markers of pre-synaptic boutons, bassoon and vesicle-associated membrane protein 2, were used. The majority of these varicosities in rcTBI mice did not contain the two pre-synaptic markers and were significantly larger than pre-synaptic boutons in size (Gu et al., 2017). Thus, most of these varicosities induced in rcTBI mice were not likely pre-synaptic boutons. Of note, there were many axons in the cortex without any varicosity or other clear morphological change. Collectively, this experimental result confirmed that axonal varicosities indeed rapidly form upon mechanical impact *in vivo* in the mouse model of repetitive mTBI, suggesting that at least some axonal varicosities were induced by the primary injury in mTBI. Since 2018, several groups independently reported that axonal varicosities were induced from a few hours to 2 days after impact in the brain of different mTBI mouse models (Marion et al., 2018; Ziogas and Koliatsos, 2018; Pernici et al., 2019; Weber et al., 2019), consistent with the findings using rcTBI.

By combining the closed-skull impact model and CLARITY imaging, the Armstrong group showed massive induction of axonal varicosities in the corpus callosum 3 days after the impact (Figure 4B), accompanied by slowed propagation of compound action potentials across the region (Marion et al., 2018). Ultrastructurally, dispersed demyelinated axons and disorganized myelin attachment to axons at paranodes were apparent within corpus callosum (Figure 4C) (Marion et al., 2018). These results are consistent with the fact that TBI patients often exhibit slowed information processing speed that can underlie diverse symptoms. Interestingly, a follow-up study from the same group showed that axonal damage including varicosity formation at 3 days post-impact dramatically reduced in SARM1^−/−^ mice, as compared to the wild-type control mice (Marion et al., 2019). SARM1 (sterile alpha and toll/interleukin-1 receptor motif-containing 1) is an evolutionarily conserved protein and functions down-stream of nicotinamide mononucleotide adenylyl transferase 2 (NMNAT2) to hydrolyze NAD^+^, resulting in depletion of NAD^+^ axonal levels and initiation of axon degeneration (Gerdts et al., 2016; Essuman et al., 2017). At the same time, it is important to note that global genetic deletion of SARM1 had rather complicated effects. While reducing mTBI-induced axonal varicosities and demyelination, SARM1 deletion itself also resulted in larger axons with thinner myelin and increased corpus callosum astrogliosis (Marion et al., 2019).

In a weight drop model combined with Thy1-YFP transgenic mice and CLARITY, the Koliatsos group reported axonal pathology including axonal varicosities induced 3 h post injury in multiple axonal tracts (Figure 4D) (Ziogas and Koliatsos, 2018). Here injury was produced by dropping a brass weight from a height of 1 m through a plexiglass tube onto a disk attached to the mouse skull, and then mouse brains were fixed 3, 24, or 48 h later for imaging analysis (Ziogas and Koliatsos, 2018). This study mainly focused on the corticospinal tract by visualizing the entire tract from the pons to the cervical spinal cord in 3D and counting the total number of axonal lesions and their progression over time. The findings showed progressive traumatic axonopathy along with blood–brain barrier disruption and neuroinflammation, and importantly axonal varicosities (Ziogas and Koliatsos, 2018). Interestingly, using genetic deletion or pharmacological inhibition of SARM1 to block the NAD^+^-related axonal self-destruction pathway, the study reported a significant reduction in the number of axonal lesions early after injury (Ziogas and Koliatsos, 2018). Therefore, future investigations are needed to elucidate the potential key role of the SARM1-NAD^+^ pathway in mTBI-induced axonal injury in order to identify an effective treatment.

Axonal varicosity formation was also observed from open-skull impact TBI models. Using a central fluid percussion injury (FPI) model, the Povlishock group showed that axonal varicosities in the optic nerve formed as early as 5–15 min post injury, and appeared to associate with an accumulation of mitochondria under electron microscopy (Wang et al., 2011). By combining the FPI model and GABAergic interneuron markers, the same group reported that axonal varicosities were developed 3 h post injury in the cortex (Figure 4E) (Vascak et al., 2018). Thus, axonal varicosities can form in both glutamatergic excitatory and GABAergic inhibitory neurons after mechanical impact. In all above-mentioned mTBI models, axonal morphology could only be compared between different mice—the ones received mechanical impact and the sham. Consistency of histology, lesion quantification and statistical analysis across different brain regions is very important to extract reliable results from many different mice.

By combining the FPI model and *in vivo* imaging using high-resolution gradient index lens technology, the Murray group was able to examine axonal changes in the same mouse and visualized axonal morphology from external capsule in both pre-injury and post-injury stages for up to 60 days after injury. They found axonal varicosities and undulated axons 1 h post injury (Pernici et al., 2019, 2020). Administration of minocycline to reduce microglia activation for three days starting 1 or 72 h after injury inhibited axonal varicosity formation or promoted axon recovery, respectively (Pernici et al., 2020). Despite clear advantages, the *in vivo* imaging often involves extensive experimental procedure in order to examine a limited number of axons in a highly focused brain regions. It increases the difficulty if axonal injury is in a multifocal pattern and restricted in a small number of axons. Moreover, the lens probe for *in vivo* imaging may also complicate the local biomechanical property for the brain region to be visualized. Thus, to develop the next generation of *in vivo* imaging that overcomes these problems will greatly advance our understanding of the development of primary and secondary injuries in mTBI.

Combining CLARITY with controlled cortical impact model of TBI, an open-skull impact model, in which the metal piston tip directly contacts with the dura of mouse brain, the Johnson group reported axonal varicosity formation 6 h after impact (Weber et al., 2019). Different from above-mentioned TBI models, this study did not use any fluorescent-protein-labeled transgenic mouse line, but used APP immunostaining to label injured axons. The morphology and connection status of APP+ swellings in peri-contusional cortex and underlying corpus callosum and hippocampus, were imaged using CLARITY at 6, 24 h, 1 week, and 1 month following the impact. These swellings first appeared as varicosities along intact axons, and could ultimately undergo secondary disconnection to become retraction bulbs (Weber et al., 2019). This study has noted that (1) confocal image stacks were needed to clearly visualize the narrow axonal shaft linking two adjacent varicosities, in order to distinguish them from retraction bulbs of broken axons, and (2) some axonal varicosities must have recovered based on the quantification (Weber et al., 2019).

Taken together, axonal varicosities may form during both primary and secondary injuries, whereas retraction bulbs appear to more likely form during secondary injury. However, it remains unclear whether some retraction bulbs can form in the primary injury. Consistent with the results from cultured neurons (Gu et al., 2017), axonal varicosities can recover *in vivo* as suggested by direct visualization using *in vivo* imaging, as well as implied by reduced varicosities and animal behavioral recovery days after impact.

### Regulation of Axonal Varicosities

Mechanical stress is not the only way to induce axonal varicosities. Microtubules are intrinsically polarized due to the head-to-tail arrangement of α/β tubulin dimers and vital for directed and long-distance transport inside neurons (Barry and Gu, 2013). Microtubule assembly-disassembly dynamics is tightly regulated by post-translational modifications and their binding proteins *in vivo* (Terada and Hirokawa, 2000; van Beuningen and Hoogenraad, 2016). Microtubule-stabilizing agents are being developed as potential therapeutics for cancer and neurodegenerative disorders (Magen and Gozes, 2013; Brunden et al., 2014; Lou et al., 2014). Microtubule breakage was implicated in DAI in mTBI or concussion (Tang-Schomer et al., 2012; Johnson et al., 2013; del Mar et al., 2015; Chuckowree et al., 2018; Cross et al., 2019). Microtubule-associated protein tau forms pathological aggregates in mTBI-induced chronic traumatic encephalopathy, while microtubule disruption is believed to underlie tauopathies (Terrell et al., 2008; Abrahams et al., 2019; Derry et al., 2019; Katsumoto et al., 2019).

Nocodazole-induced microtubule depolymerization can directly induce axonal varicosity formation from primary cultured CNS neurons without mechanical stress (Gu et al., 2017). To determine whether actin or microtubule cytoskeleton is involved in axonal varicosity initiation, cultured neurons were treated with 10 μg/ml nocodazole (depolymerizing microtubule filaments) or 2.5 μM latrunculin A (depolymerizing actin filaments). Nocodazole (for 15 min) but not latrunculin A (for up to 150 min) treatment induced axonal varicosities (Gu et al., 2017). Of note, different from fluid puffing, it took much longer time for nocodazole (at the concentration) to induce axonal varicosities. The treatment also induced dendritic varicosities at the same time, different from fluid puffing, which induced varicosities only in axons (Gu et al., 2017). Microtubule disruption appears to be part of the signaling pathway for varicosity induction downstream of mechanical stress induced Ca^2+^ influx.

Mitochondria dysfunction can lead to irreversible axonal varicosity formation followed by degeneration. Mitochondria depend on a proton gradient to generate ATP and disrupting electron transport chain function can result in the deleterious accumulation of ROS. This process has been linked to the pathogenic mechanisms underlying many neurological disorders (Lin and Beal, 2006). For instance, mitochondrial depolarization by protonophore treatment leads to aberrant Ca^2+^ influx, axon blebbing and fragmentation before cell death (Summers et al., 2014). Inhibition of electron transport chain complex I and increased ROS production by hydrogen peroxide can also lead to axon degeneration, which can be protected by SARM1 deletion, whereas SARM1 appeared to work downstream of Ca^2+^ influx (Summers et al., 2014). Different from mechanical stress-induced axonal varicosities, axonal degeneration is irreversible.

Action potentials were shown to alter axonal morphology (Cohen, 1973; Iwasa et al., 1980). Using time-lapse super-resolution microscopy, a recent study reported that high frequency firing can transiently enlarge (up to 20%) axonal varicosities—synaptic boutons—of hippocampal CA3 neurons, and increase the diameter (up to 6%) of axonal shafts in a relatively longer term (Chereau et al., 2017). The experiments were performed with a STED microscope for live-cell imaging of GFP-labeled axons in organotypic brain slices (Chereau et al., 2017). Ca^2+^ entry appeared critical for the initial enlargement of the boutons, since Cd^2+^ (blocking Ca^2+^ channels) abolished the enlargement. Both simulation and electrophysiological recording revealed a phase of slowed down action potential conduction linked to the transient enlargement of the synaptic boutons, followed by a sustained increase in conduction speed accompanying the axon shaft widening, in response to high-frequency action potential firing (Chereau et al., 2017). This fine tuning of axonal morphology differs from mechanical stress-induced axonal varicosities in *de novo* induction from smooth axonal shafts and changing size. The diameter of mechanical stress-induced axonal varicosities can reach up to ten times of that of the adjacent axonal shafts. Nonetheless, regulation of pre-synaptic boutons also involves Ca^2+^ and microtubules, just like those induced axonal varicosities. It will be interesting to determine whether micromechanical stress plays a key role in synaptic formation and maintenance, and whether synaptic boutons can retract similar to the recovery of axonal varicosities. These are potentially important questions in order to gain a better understanding of wiring and rewiring of neural circuits in the CNS.

### Future Perspective

Axonal intrinsic mechanisms governing varicosity formation induced by mechanical stress remain to be fully understood. The first important question is whether axonal varicosities can be effectively and equally induced by different types of mechanical stresses, such as compression, tension (or stretch), shear, bending and torsion. The answer to this question will shed light on the potential specificity of a distinct mechanosensing signaling pathway to a specific type of stress. Although limited progress was made to understand the roles of mechanosensitive ion channel (TRPV4) and microtubule stability (via MAP6) in mechanosensation (Gu et al., 2017), much more efforts are still needed to investigate the signaling pathway(s) involved, for instance the potential roles of other types of mechanosensitive ion channels and membrane proteins. Another unknown aspect is the potential dynamic changes of local osmolarity during the development of axonal varicosities.

Extrinsic mechanisms underlying axonal varicosity formation induced by mechanical stress remain to be elucidated. A neuron cannot properly function alone, and it has to physically interact with its microenvironment, in addition to chemical and electrical communications. So far, it remains largely unknown how myelinating oligodendrocytes, astrocytes, microglia or blood vessels regulate mechanical stress-induced axonal varicosity formation directly or indirectly through released factors. The beads-on-a-string shape of induced axonal varicosities resembles some pre-synaptic axonal boutons, raising an intriguing question whether the regulatory mechanisms of synaptic formation can also affect induced axonal varicosities. Conversely, a better understanding of axonal varicosity formation may provide new mechanistic insights into pre-synaptic initiation and development. Therefore, rapid and reversible formation of axonal varicosities in CNS neuron mechanosensation is likely a newly-recognized form of neural plasticity and has broad implication in neurobiology.

Enhancing the recovery of axonal varicosities may become a key strategy to restore the structure and function of stressed or injured axons. Axonal varicosity formation appears to be an early event in axonal primary injury in mTBI. It is also possible that the secondary injury in mTBI can induce axonal varicosity formation through a different signaling pathway. Therefore, it is important to not only understand the heterogeneity of axonal varicosities but also link these differences to the primary or secondary injury. These insights will provide valuable information for developing new strategies to enhance the recovery of axonal varicosities, in order to reverse axon pathology in treating mTBI and perhaps related neurological diseases as well.

## Author Contributions

The author confirms being the sole contributor of this work and has approved it for publication.

## Conflict of Interest

The author declares that the research was conducted in the absence of any commercial or financial relationships that could be construed as a potential conflict of interest.
